# Supplementary material for: Hormone circuit explains why most HPA drugs fail for mood disorders and predicts the few that work
Source: Mol Syst Biol. 2025 Jan 23;21(3):254–73. doi: 10.1038/s44320-024-00083-0 (PMC11876312; doi:10.1038/s44320-024-00083-0)
Supplement: Supplementary file 2 — Expanded View Figures [file 44320_2024_83_MOESM2_ESM.pdf]

Expanded View Figures

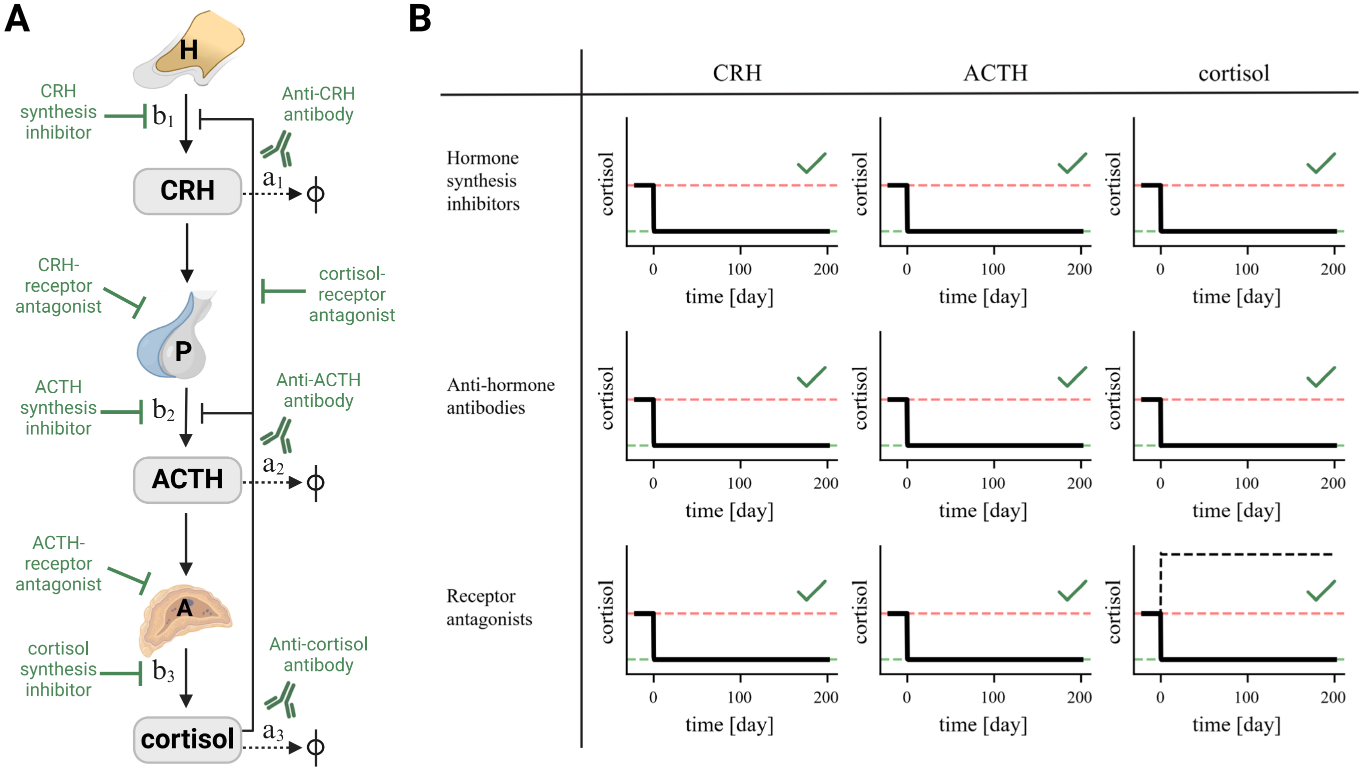

Figure EV1. Efficacy of potential interventions according to a HPA model with nonadjustable glands, related to Fig. 1.

Panels similar to Fig. 1. (**A**) The classical HPA model with nonadjustable glands. (**B**) Simulations of HPA-modulating drugs' effect on cortisol levels under the classical HPA model.

 © The Author(s)

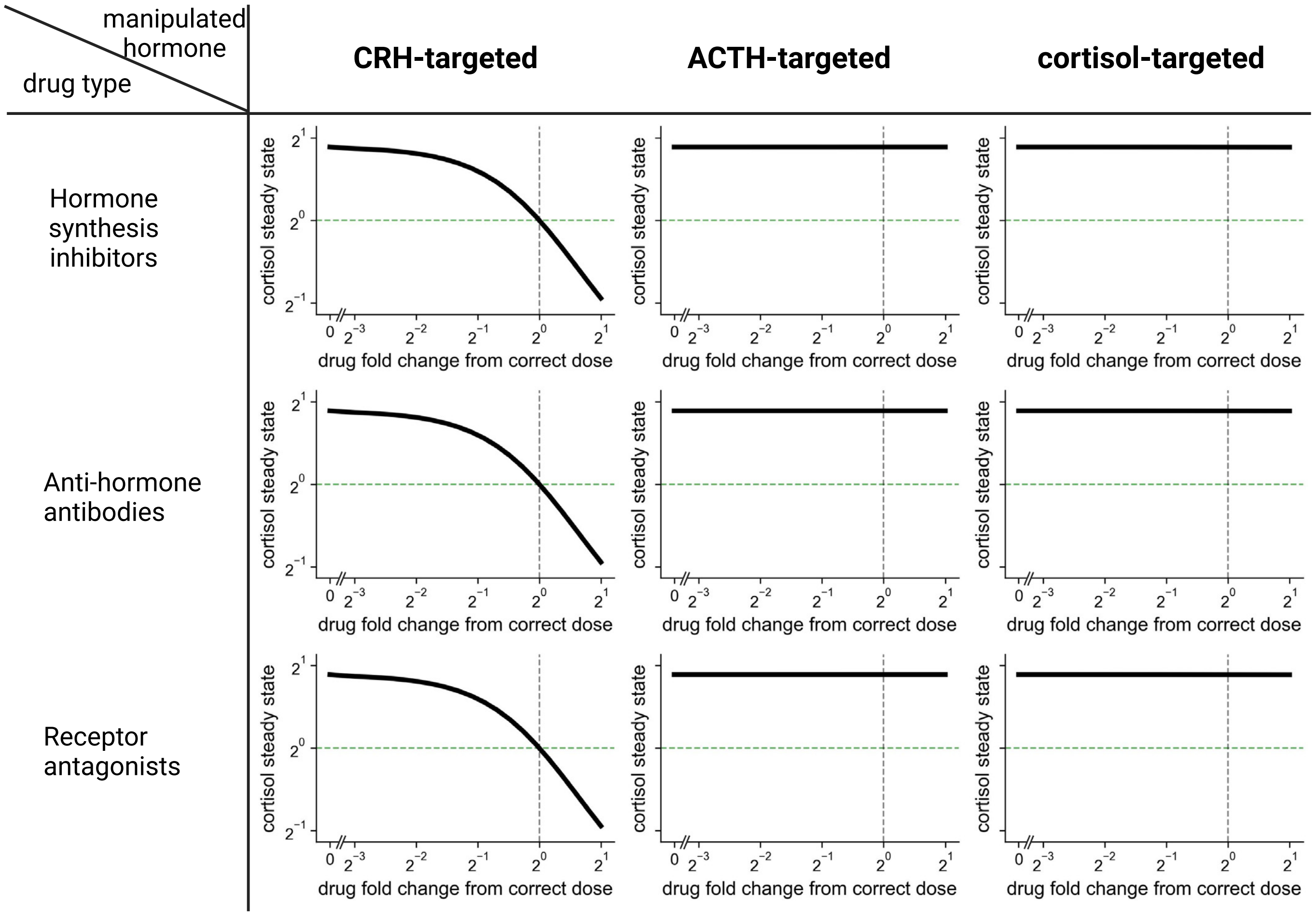

Figure EV2. Steady-state cortisol dose response to HPA-targeting drugs, related to Fig. 4A.

Cortisol steady state in the model upon changes in doses of HPA-targeting drugs. Horizontal dashed green lines indicate normal cortisol steady-state level; Vertical dashed gray lines indicate the drug dose that normalizes cortisol.

© The Author(s)

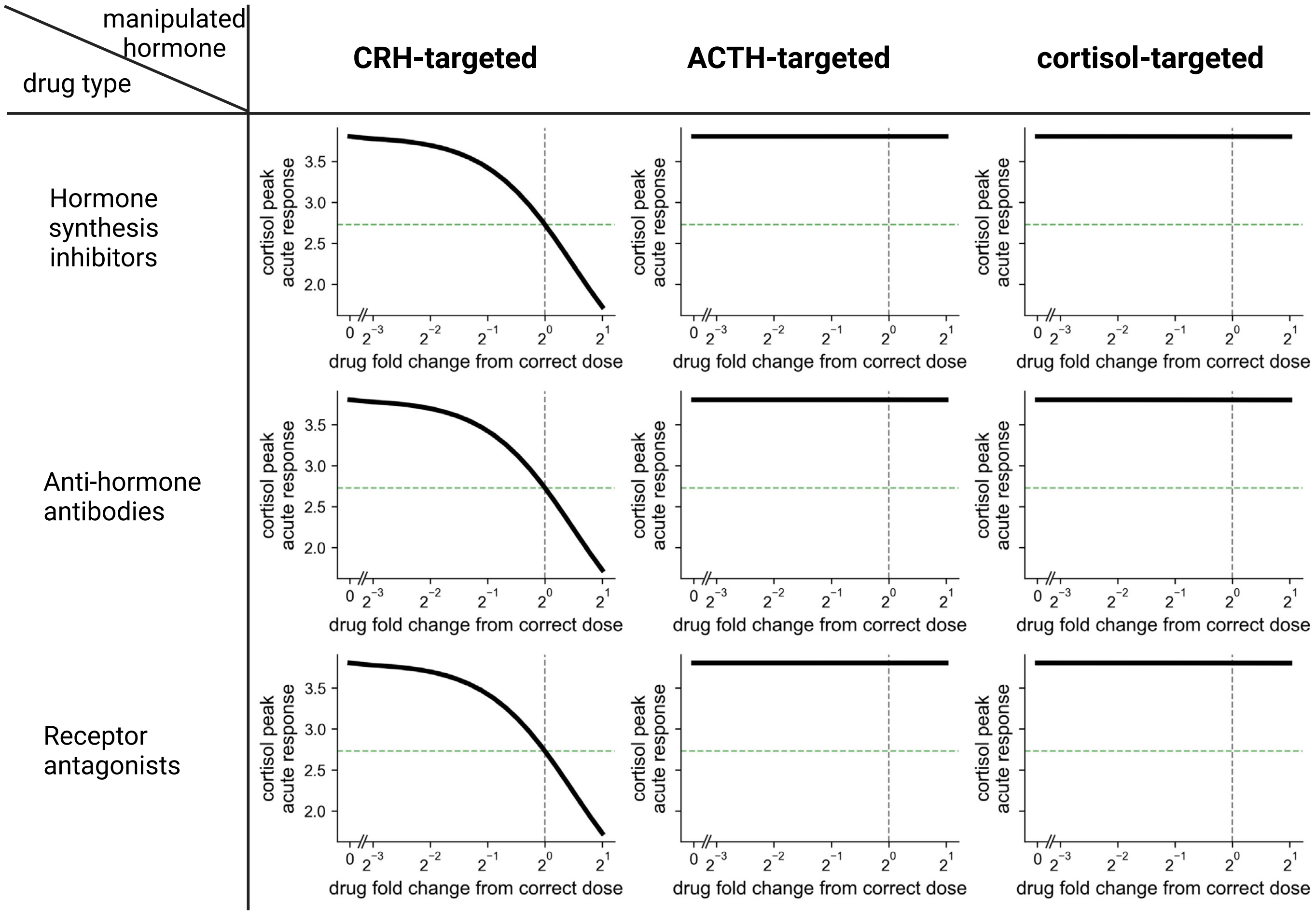

Figure EV3. Cortisol peak response to acute stressor under varying concentrations of HPA-targeting drugs, related to Fig. 4D.

Cortisol peak response to acute stress relative to steady state for different doses of HPA-targeting drugs. Horizontal dashed green lines indicate normal response; Vertical dashed gray lines indicate the drug dose that normalizes the response.

 © The Author(s)

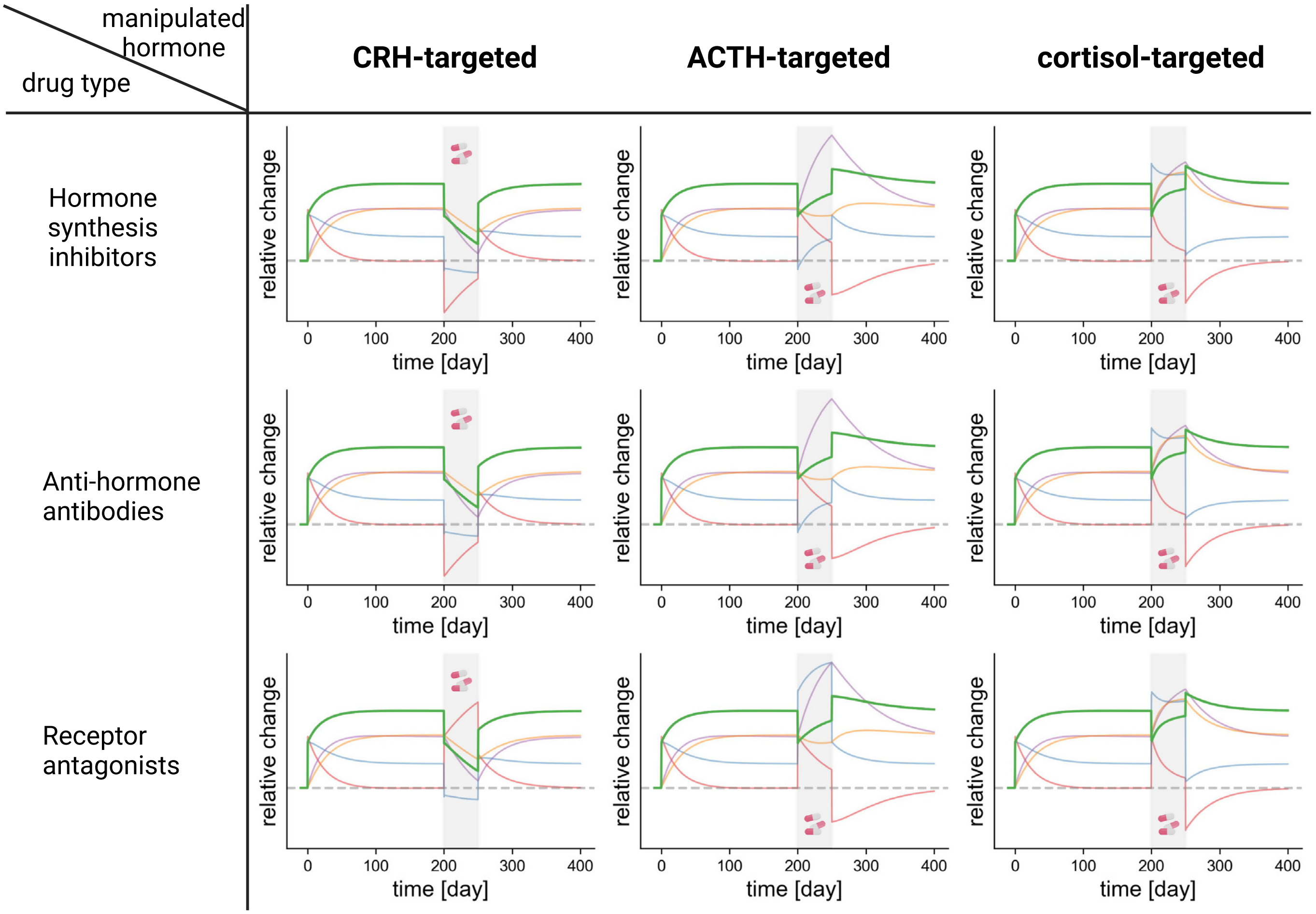

Figure EV4. Full HPA dynamics upon treatment cessation, related to Fig. 4E,F.

HPA dynamics upon cessation of HPA-targeting drugs after 50 days. For color legend see Fig. 4E,F.

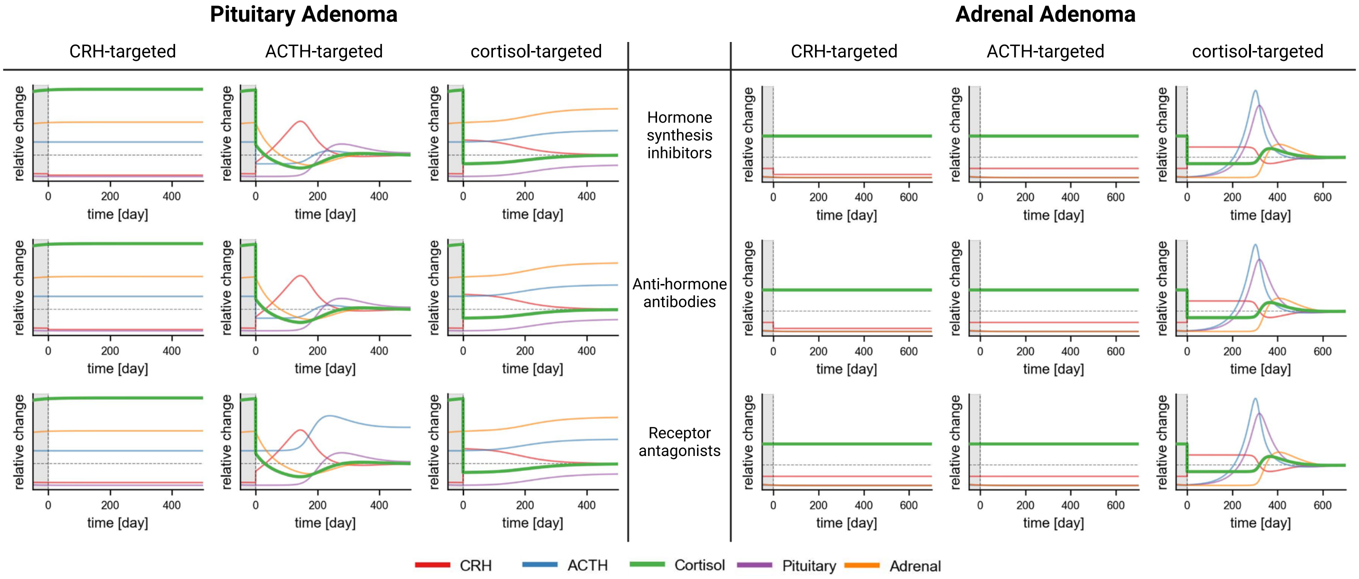

Figure EV5. HPA dynamics under HPA-targeting drugs in Cushing syndrome, related to Fig. 5.

Simulations of the HPA dynamics during treating Cushing syndrome caused by a pituitary adenoma (left) or by an adrenal adenoma (right). The simulation starts with untreated Cushing (gray shaded region) and at point zero the simulated drug is administered.

 © The Author(s)
